# Development of a Nuclear Magnetic Resonance Method and a Near Infrared Calibration Model for the Rapid Determination of Lipid Content in the Field Pea (*Pisum sativum*)

**DOI:** 10.3390/molecules27051642

**Published:** 2022-03-02

**Authors:** Philip Wiredu Addo, Philip Ossowski, Sarah MacPherson, Andrée E. Gravel, Rajvinder Kaur, Valerio Hoyos-Villegas, Jaswinder Singh, Valérie Orsat, Marie-Josée Dumont, Mark Lefsrud

**Affiliations:** 1 Bioresource Engineering Department, McGill University, Macdonald Campus, Ste-Anne-de-Bellevue, QC H9X 3V9, Canada; philip.addo@mail.mcgill.ca (P.W.A.); ptossowski@gmail.com (P.O.); sarahanne.macpherson@mcgill.ca (S.M.); rajvinder.kaur@mcgill.ca (R.K.); valerie.orsat@mcgill.ca (V.O.); marie-josee.dumont@mcgill.ca (M.-J.D.); 2 Drug Discovery Platform, Research Institute McGill University Health Centre, Montreal, QC H4A 3J1, Canada; andreegravel@hotmail.com; 3 Plant Science Department, McGill University, Macdonald Campus, Ste-Anne-de-Bellevue, QC H9X 3V9, Canada; valerio.hoyos-villegas@mcgill.ca (V.H.-V.); jaswinder.singh@mcgill.ca (J.S.)

**Keywords:** fatty acid, legume, near infrared, nuclear magnetic resonance, phytochemicals, *Pisum sativum*

## Abstract

*Pisum sativum* is a leguminous crop suitable for cultivation worldwide. It is used as a forage or dried seed supplement in animal feed and, more recently, as a potential non-traditional oilseed. This study aimed to develop a low-cost, rapid, and non-destructive method for analyzing pea lipids with no chemical modifications that would prove superior to existing destructive solvent extraction methods. Different pea accession seed samples, prepared as either small portions (0.5 mm^2^) of endosperm or ground pea seed powder for comparison, were subjected to HR-MAS NMR analyses and whole seed samples underwent NIR analyses. The total lipid content ranged between 0.57–3.45% and 1.3–2.6% with NMR and NIR, respectively. Compared to traditional extraction with butanol, hexane-isopropanol, and petroleum ether, correlation coefficients were 0.77 (R^2^ = 0.60), 0.56 (R^2^ = 0.47), and 0.78 (R^2^ = 0.62), respectively. Correlation coefficients for NMR compared to traditional extraction increased to 0.97 (R^2^ = 0.99) with appropriate correction factors. PLS regression analyses confirmed the application of this technology for rapid lipid content determination, with trends fitting models often close to an R^2^ of 0.95. A better robust NIR quantification model can be developed by increasing the number of samples with more diversity.

## 1. Introduction

For more than 50 years, society has depended on the organic chemistry industry to produce basic necessities, such as food, pharmaceuticals, and chemicals. Today, 92% of organic products are synthesized in the petrochemical industry using either crude oil or natural gas. Recent issues related to climate change, due to the increase in greenhouse gases, as a result of the petrochemical industry, has led to more research into bio-products.

Although legume or pulse crops are mostly cultivated to enhance protein content for human consumption and animal feed, it may be possible to improve lipid concentration through cross-breeding and genetic engineering to increase their value as enhanced bio-products. Legume protein content ranges from 25–32% [1,2] and lipid content ranges between 0.9–46% [3,4].

Field pea (*Pisum sativum*) is an important pulse crop that is mostly grown in temperate regions. It is used as either forage or a dried seed supplement in animal feed, and more recently, has been considered, along with other pulse crops, as a potential non-traditional oilseed crop that can help meet global demands for vegetable oil and biodiesel [5]. In addition, field peas are a good source of protein, carbohydrates, phenolic compounds, phytic acid, fiber, lipids, tannins, and amino acids [6]. Peas contain approximately 87% total digestible nutrients, increasing the nutritional value of most diets [7].

Lipids are comprised of fatty acids that are present in the endosperm of the field pea seed [8]. Fatty acid composition is influenced by agro-climatic conditions as the enzymes in fatty acid biosynthesis depend on these factors [9]. Fatty acid content ranges between 0.9–5.0% of dry matter [5]. The low quantity of saturated fatty acids found in peas in comparison to unsaturated fatty acids can be explained by the conversion of the polyunsaturated fatty acids to carbohydrates by lipid peroxidation [10]. This is because the fatty acids contain multiple double bonds and the methyl group that lies within is prone to hydrogen atom abstraction. Research is ongoing to increase the lipid content in field pea using genetic engineering and traditional breeding [11].

Various studies have been conducted using traditional methods for extraction and quantification of lipids, as well as for fatty acid profiling of plant material [12,13,14]. Traditional methods for fatty acid determination involve triacylglycerol hydrolysis, followed by the esterification of the liberated fatty acyl groups with methanol or by direct transesterification and, finally, gas chromatography to separate, identify and quantify fatty acids that are present. This process is required because the majority of the fatty acids exist in the form of esters and amides, and only a small portion constitutes free fatty acids. These traditional methods are labor-intensive and time- and chemical-consuming. They expose the oil to lipid oxidation and the process requires fatty acid standards [15]. Previous solvent extractions have demonstrated percentage lipid content in pea samples ranging from 1.3% ± 0.07% to 2.6% ± 0.11% [16]. More recently, fatty acid determination has evolved with methods that allow for qualitative and quantitative analyses. Modern methods include nuclear magnetic resonance (NMR) and near infrared (NIR) spectroscopies, where spectroscopy is defined as the interaction between matter and electromagnetic radiation.

NMR is based on the absorption of electromagnetic radiation in the radio frequency region by atomic nuclei [17]. This technology has been studied for decades and it has become an important and powerful analytical tool used by researchers [18]. NMR has applications in a wide range of disciplines such as the food, pharmaceutical, chemical and petroleum industries, as well as the agricultural and disease control sectors. In the pharmaceutical industry, it is a useful technique for drug discovery and determining the conformations of the compounds bound to enzymes, receptors, and other proteins [19]. In the agricultural and food industries, NMR is mostly used for quantitative and qualitative analyses, such as structure determination, chemical composition, product purity and adulterations, kinetic reaction monitoring, in situ tests for ripening, etc. NMR can be used to compare the different profiles of genetically modified foods to their unmodified counterparts [20].

NIR spectroscopy is based on the region of the electromagnetic spectrum between visible light and infrared radiation, occurring from approximately 780 nm to 2600 nm [21]. It involves changes in vibration energies of the molecular bonds O-H, C-H, C-O, and N-H when subjected to NIR frequencies, wherein bonds undergo vibrational stretching and bending [22]. Calibration methods have been developed using NIR spectroscopy for rapid determination of seed contents, including lipids, starch, carbohydrates, proteins, moisture, fatty acid composition, phytonutrients, and many other components [23,24]. In the food industry, NIR is used to determine variations in raw materials and finished food products due to different processing procedures [25]. This study aimed to investigate how modern spectroscopy methods, NMR and NIR, could be used to study fatty acids in pea, while developing calibration models to predict pea lipid content in this non-traditional oilseed. Data presented here could advance the development of a low-cost, rapid, and non-destructive method for lipid profiling and determining lipid content in leguminous crops that is superior to traditional destructive solvent extraction methods.

## 2. Results and Discussion

### 2.1. HR-MAS NMR Spectroscopy

**Figure 1:**
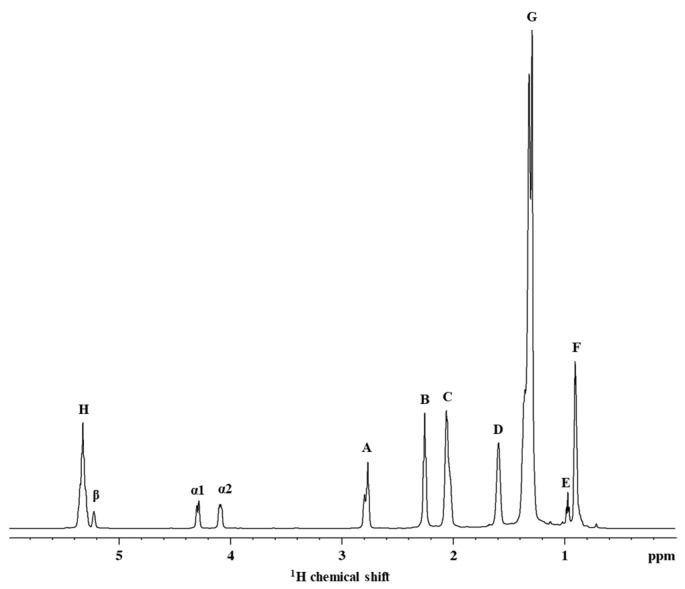
^1^H NMR spectrum of the pea oil sample.

A review of the literature shows that the NMR spectrum of the pea is similar to those of various oilseeds analyzed using ^1^H high-resolution magic angle spinning (HR-MAS) NMR under similar conditions [26,27,28]. The ^1^H NMR spectrum of the pea oil contains 11 distinct signals of variable intensity (Figure 1). Different signals observed in the spectrum are a result of protons in the same chemical environment, where neighbouring protons cause peak splitting. However, the intensity of the signal depends on the proportion of different acyl groups in any given sample [26]. The use of the magic angle spinning property of the HR-MAS probe was efficient at averaging residual dipolar interactions and variations in the bulk magnetic susceptibility of the samples. NMR spectra displayed a resolution approaching that of liquid samples. Some samples achieved lower spectral resolution than others where the splitting of multiplets could not be distinguished.

Signal A (=HC-CH_2_-CH=) represents bis-allylic hydrogen atoms and is caused by overlapping signals from α-methylene protons in two double bonds. Bis-allylic hydrogens are used to determine the nature of the unsaturated fatty acids present in each sample. Signals B (-OCO-CH_2_-) and D (-OCO-CH_2_-CH_2_-) represent methylene hydrogen atoms in the β and α positions in relation to the carbonyl group, respectively. Signal C (-CH_2_-CH=CH-) represents allylic hydrogen atoms, caused by α methylene hydrogen atoms in a single double bond (allylic carbon). All methyl protons in saturated or unsaturated acyl groups containing a double bond further from the terminal methyl group give rise to a triplet signal (Signal F). Signal F (-CH_3_) is produced by the overlap of triplet signals from the methyl group protons of saturated, oleic acyl and linoleic acyl groups [26,27,28].

Signal E (-CH_3_) represents the methyl protons of ω-3 acyl groups. The difference in chemical shifts between methyl proton signals is due to their proximity to the double bond of the fatty acid chain. Signal G (-(CH_2_)n-) represents the hydrogen atoms of those methylene groups, either in position β or further from the olefinic groups, or in position γ or further from the carboxylic groups inside the triglyceride molecule. The α hydrogens of the glycerol backbone on carbons 1 and 3 cause signals α1 and α2 (-CH_2_OCOR). The β (-CHOCOR) signal is due to the β hydrogen atom on carbon 2 of the glycerol backbone. Lastly, signal H (-CH=CH-) overlaps slightly with signal β due to the olefinic hydrogen atoms of the different acyl groups [26,27,28].

#### 2.1.1. Fatty Acid Composition of Pea Seeds Using ^1^H HR-MAS NMR

The observed chemical shifts of the signals were not significantly (Figure 1) (*p* > 0.05) different from those reported in the literature [26,27,28]. Importantly, data indicate that the different levels of fatty acid types are not affected by sample preparation into small pea seed portions or as ground pea seed powder (Table 1). The linoleic acid content of the tested pea seed varieties ranged between 13.89–43.98%. Oleic acid, a monounsaturated omega-9 fatty acid, was detected in all field pea varieties tested; the highest content measured was 55.42% (Sample 29579) and the lowest content measured was 21.71% (Sample 42819). Linolenic acid, an essential fatty acid, belongs to the group of omega-3 fatty acids and is highly concentrated in most plant oils. It reportedly inhibits prostaglandin synthesis and reduces the risk of chronic diseases [29]. In this study, the highest linolenic fatty acid content measured was 26.62% (Sample F1) and the lowest content measured was 20.81% (Sample 45760). Saturated fatty acids most commonly found in field peas are arachidic, behenic, palmitic, stearic, and lignoceric fatty acids, with palmitic acid being the most dominant [30]. In this study, saturated fatty acids ranged between 4.61–28.24%. The results are similar to published data [4,8].

#### 2.1.2. Quantification of Total Lipid Content Using ^1^H HR-MAS NMR

Non-extraction methods for determining total lipid content are indirect methods based on the measurement of a physical or chemical property of a sample. This includes density, dielectric measurements, NIR spectroscopy, NMR spectroscopy, ultrasound, colorimetry and X-ray absorption [31]. Extraction methods are based on the water insolubility property of lipids. This is achieved by using a solvent that is highly lipid-soluble, and less or insoluble for other cell components such as proteins and carbohydrates [32]. Diethyl ether, pentane and methylene chloride are some of the solvents commonly used for extraction of lipids from pea seeds.

Analysis of variances for different extraction methods showed no significant differences (*p* > 0.05) between Soxhlet extraction with hexane-isopropanol and butanol solvents. In contrast to petroleum ether, significant differences (*p* > 0.05) existed when a larger amount of lipid was extracted with the hexane-isopropanol and butanol solvents. This is because non-polar lipids exhibit better solubility in hexane-isopropanol and butanol [33]. In addition, these data indicate that a larger fraction of the lipid concentration in pea seeds is non-polar. In Table 2, quantification by both NMR and Soxhlet extraction with petroleum ether underestimated the total lipid content present in field pea seeds compared to Soxhlet extraction with hexane-isopropanol and butanol solvents.

**Figure 2:**
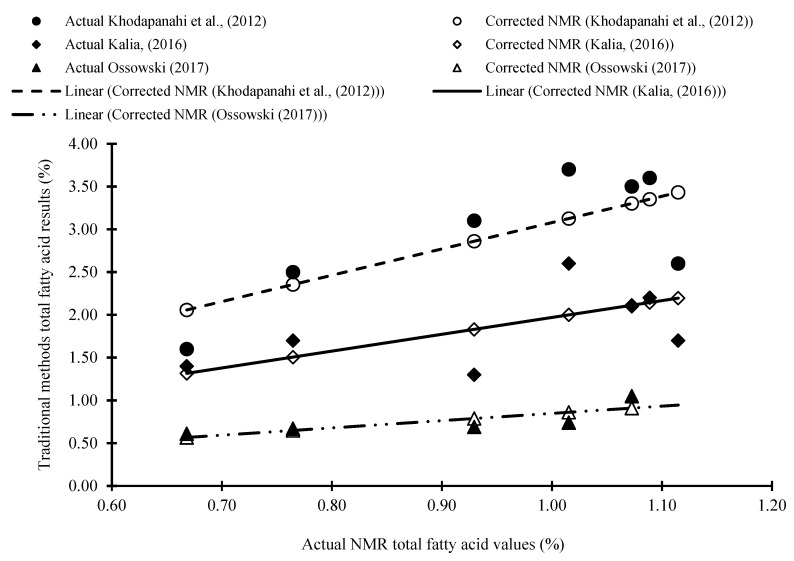
Comparing of the actual results of the traditional methods to the actual and corrected results of the NMR of field pea.

The NMR method employed in this study is rapid and non-destructive compared to traditional solvent extraction methods, and a correlation between the NMR method and other traditional methods was observed. Correlation coefficients of 0.77 (R^2^ = 0.60), 0.56 (R^2^ = 0.47), and 0.78 (R^2^ = 0.62) were observed in the Khodapanahi et al. (2012), Kalia (2016), and Ossowski (2017) studies, respectively (Table 2). Correlation coefficients were determined by comparing the results from the NMR and the three studies. The correlation coefficients for each method increased to 0.97 (R^2^ of 0.99) when NMR data were corrected with respect to the other methods using a correction factor, namely 3.08, 1.97 and 0.85 for Khodapanahi et al., (2012), Kalia, (2016), and Ossowski, (2017) respectively (Figure 2). The correction factor used was the average ratio of the data acquired for each traditional method to the NMR data. Statistical analyses showed no significant differences (*p* > 0.05) between the corrected NMR data for NMR and the corresponding quantitative data acquired with traditional methods. This demonstrates that NMR can effectively quantify total lipids in field pea seeds.

The NMR method was used to quantify lipids in soybean to validate the model. Soybean pieces were tested and a total lipid content of 4.60% was recorded by NMR (Table 2). The total lipid content was 14.17% after the correction factor was applied, which was not significantly different when compared to total lipid content determined using the Soxhlet extraction with butanol at 13.90%.

#### 2.1.3. Seed Viability

Seed viability was verified by subjecting all remaining pea seed portions from each variety to a germination test. The germination rate for all pea seeds from each variety (27 in total) was 100% (Table S1). Successful germination was as defined the emergence of the radicle (day four), plumule (day five), foliage leaves (day seven), and secondary roots (day eight). These data indicate that this NMR sampling method may be used for the non-destructive analytical testing of oilseed lipids.

### 2.2. NIR Spectroscopy

The entire spectrum of absorbance was used to generate the partial least squares (PLS) model (Figure S3) for the near infrared analyses. The PLS was calculated over the selected wavelength range of 1100 nm–2500 nm [21,34]. The near infrared spectra were pre-treated mathematically and derivatized. PLS regression generated a set of b-vector coefficients for each of these points; they were used as coefficients for calculating new predictions from similarly treated spectra. By the nature of the PLS model, quantification of lipids in each pea seed sample was achieved by using spectral features in the entire range from 1100 nm–2500 nm, rather than using the individual peaks or regions in the spectrum.

From the PLS regression analyses (Table 3), different approaches for pre-processing data were assessed for the ability to produce models that best fit the acquired data. Results describing the validation models produced are summarized in Table 3. When using NIR spectroscopy for oil determination in pea seeds [21], the use of PLS regression is recommended over other multivariate tests, such as principal component regression. Here, the effectiveness of PLS is demonstrated through the coefficient of determination (R^2^) and the standard error of cross-validation (SECV), which is the error of cross validation when corrected for bias using the CAMO Unscrambler^®^ X Software (CAMO Analytics, Oslo, Norway).

**Figure 3:**
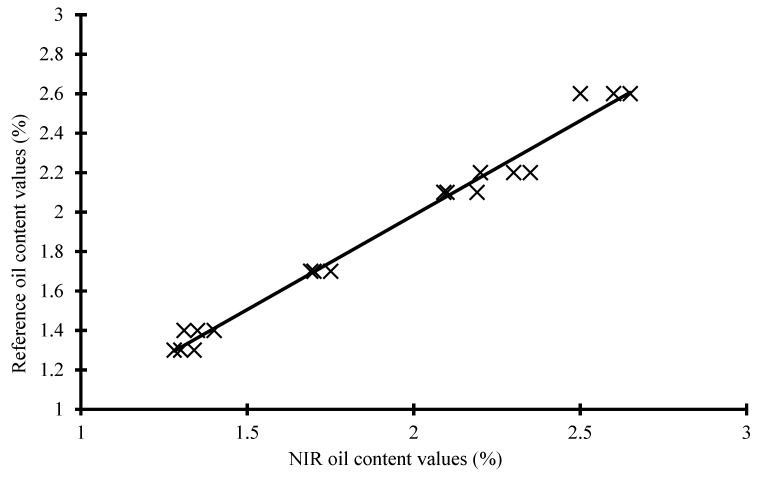
Graph of predicted (NIR) versus reference values demonstrating fitness of the model.

A basic PLS regression analysis of the data without manipulation beyond conversion to absorbance demonstrates a four-factor model with a reasonable SECV of 0.13 (R^2^ = 0.96). Improvement to the model’s fitness is demonstrated when the data is pre-processed. An interesting metric of the effectiveness of a model is the calculation of RPD, or the ratio of standard error of performance to standard deviation. RPD is calculated as the ratio of the standard derivative to the SECV. RPD values can be used to predict the approximate suitability of data for use at different levels. The higher the RPD, the more reliable is the calibration. The model is suitable for rough scans and general approximation of oil content as the values approach or exceed 4.5 RPD. There is no doubt that the models produced are insufficiently robust, as only 18 samples were used for the study. Performance of this caliber with such a small sample set would suffer from over-fitting. Modest results using NIR for component quantification with larger sample counts have reported lower RPD scores [35]. A greater number of samples would lend to a more robust model. The overall deviation between the reference values versus the calibration and validation models is seen in Figure 3.

## 3. Materials and Methods

### 3.1. HR-MAS NMR Spectroscopy

#### 3.1.1. Pea Accessions and Sample Preparation

Pea (*Pisum sativum*) oil, soybean seeds (*Glycine max, cv*. Champion), and nine wild and domestic pea accessions catalogued as 29579, 112351, 42819, F2 (G611 x Wando), F1 (112351 × 43016), 29600, 43016, 45760, and 29526 were used in this study. Field pea seeds were harvested in 2015 from the Harbin Seed Farm (Rivercourse, AL, Canada) and McGill University’s Macdonald campus (Sainte-Anne-de-Bellevue, QC, Canada). Soybean seeds were obtained from plants grown in 2015 at the Belcan Agro Centre (Sainte-Marthe, QC, Canada).

Samples were prepared as two different forms. For pea seed portions, a small fragment (0.5 mm^2^) of the endosperm of each seed was manually removed to serve as the pea seed sample for NMR analysis. Germination tests were performed on the remaining pea seed fraction to determine viability for each variety and confirm that the method used was non-destructive. Ground pea samples were achieved by grinding dry pea seeds to a fine powder with a mortar and pestle prior to NMR analysis. Serving as the external reference for total lipid quantification, one pea oil sample was extracted from pea accession 29,526 using the Soxhlet extraction method with 12 mL hexane-isopropanol (3:2, *v*/*v*) at room temperature.

#### 3.1.2. Instrumental Setup

Fatty acid profiling and total lipid content of pea seeds were determined using the high-resolution magic angle spinning (HR-MAS) NMR equipment at the Research Institute of the McGill University Health Centre (Montreal, QC, Canada). These experiments were performed using a Bruker AVANCE III HD 600 spectrometer (Bruker Cooperation, Billerica, MA, United States) operating at 600.17 MHz for ^1^H. A 4-mm triple-resonance ^1^H/^31^P/^13^C high-resolution MAS probe with a Z-gradient directed along the magic angle axis was employed. All seed samples were spun at 5000 Hz in zirconia rotors and experiments were run at room temperature (~20 °C). Approximately 15 μL deuterium oxide (D_2_O) containing 5 mM 3-(trimethylsilyl) propionic-2, 2, 3, 3-d4 acid (D_2_O + TSP; Sigma-Aldrich, Oakville, ON, Canada) was pipetted into the bottom of the rotor to serve as an internal standard for chemical shift references. Approximately 7 mg of each sample (pea seed portion or ground sample) were placed into a Bruker KelF insert and combined with 15μL D_2_O. The insert was then closed with a plug and a sealing screw. The insert was placed in the rotor and ^1^H spectra were acquired with a 1-D pulse sequence and water pre-saturation (Bruker pulse sequence zgpr). Each spectrum was acquired with 128 scans, a 6 μs 90° pulse length, 8417.5 Hz spectral width, 16k data points, a 0.97 s acquisition time, and a 1 s relaxation delay. Solution-type NMR experiments were achieved for the study by spinning at the magic angle using the high-resolution magic angle spinning (HR-MAS) probe. Only ^1^H experiments were acquired, as is common when using HR-MAS probes. The experiment was conducted in triplicate for each accession.

#### 3.1.3. HR-MAS NMR Spectra Processing

The NMR method used for lipid quantification was determined by knowing that all fatty acid acyl chains were esterified to a common moiety, a glycerol backbone that forms a triacylglyceride [36]. The area under each signal in the proton NMR spectrum is proportional to the number of hydrogen atoms for the different types of aliphatic groups of the fatty acid chain [37]. Integration of the signals was done using Topspin 4.0.3 software (Bruker Cooperation, Billerica, MA, USA) to find the area values under the signals. Preliminary tests showed that TSP was not a reliable reference for the quantification of fatty acids. The accuracy of the TSP quantification method can be affected by the binding of TSP to fatty acids and the displacement of TSP into small spaces inside the spinning rotor that are outside of the radio frequency coil’s detectable volume [38,39]. The pea oil sample was used as the quantification reference and for normalizing the sum of the integrals to 100. The spectrum for each field pea seed sample (Figure S1) was compared to the pea oil sample using the multiple integration NMR spectra function, generating integral values (Table S2). This was used to calculate proportions of the different acyl groups in each sample using the following equations:

Linolenic fatty acid (C18:3) (%) = 100% [E/(F + E)](1)

Linoleic fatty acid (C18:2) (%) = 100% [(A/B) − 2[E/(F + E)](2)

Oleic fatty acid(C18:1) (%) = 100% [(C/2B) − (A/B) + [E/(F + E)](3)

Saturated fatty acids (%) = 100% [1 − (C/2B)](4)

where A represents HC-CH_2_-CH acyl groups; B represents -OCO-CH_2_- acyl groups; C represents -CH_2_-CH=CH- acyl groups; D represents -OCO-CH_2_-CH_2_- acyl groups; E represents a -CH_3_ linolenic acyl group; and F represents -CH_3_ saturated, oleic and linoleic acyl groups [37].

#### 3.1.4. Statistical Analyses of HR-MAS NMR Data

SAS University Edition vApp (SAS Institute Inc., Cary, NC, US) was used for data analyses. A completely random design model of two factors (variety and processed sample form) and Student–Newman–Keuls pairwise comparison was used for this study.

### 3.2. NIR Spectroscopy

Six wild pea accessions, 29526, 29579, 42819, 43016, 45760, and 112351, were tested for NIR analyses. Pea plants were field grown and harvested in the summers of 2014 and 2015 at McGill University’s Macdonald campus and dried in an oven at 40 °C for 72 h before long-term storage.

#### 3.2.1. Instrumental Setup and Scan Parameters

NIR scans were taken on a FieldSpec 4 Standard-Res portable spectroradiometer (ASD Incorporated, Longmont, CO, United States). The FieldSpec 4 was equipped with an ASD Contact Probe (ASD Incorporated, Longmont, CO, USA), a 10-mm scan spot size and a halogen bulb light source (2900 K colour temperature). Scans were taken over the full instrument spectrum of 350 nm to 2500 nm. The room where scans took place was environmentally lit with standard fluorescent lighting, rated at a colour temperature of 3500 K. The instrument was calibrated with a Spectralon fluoropolymer calibration standard with a diffuse reflectance of >95% between 250 nm and 2500 nm. Clear Petri dishes (100 mm × 15 mm) were filled and levelled with whole pea samples. The experiment was conducted in triplicate for each accession.

A turntable was designed from the rotating head of a tube rotator adjusted to sit horizontally (Figure S2). A Styrofoam tray was modified and attached to the head of the rotator to center and secure Petri dishes containing each whole pea sample. Samples were rotated at 22 rpm, with the instrument’s probe fixated approximately 30 mm from the center of each dish, 5 mm above the sample. For each sample dish, the instrument was programmed to take average reflectance values of 100 scans over a 17-s period as the sample dish rotated. Each Petri dish containing one sample was scanned and analyzed chemometrically using UCal 4^TM^ Custom Calibration Software (Unity Scientific, Milford, MA, USA).

#### 3.2.2. Data Pre-Processing

NIR spectra were analyzed and processed using UCal 4^TM^ Custom Calibration Software. A data matrix was configured to associate the reference lipid values determined previously [16] with their corresponding spectra. Reflectance spectra were converted to absorbance by taking the logarithm of the inverse of the reflectance values. Applicable wavelengths were consolidated to the NIR range of 1100–2500 nm. Standard normal variate and math treatments were performed on the full sample set to assess their ability to remove additive baseline and scattering effects. Multiplicative scattering correction was applied to reduce the effects of light scattering produced by the sampling method. This eliminated deleterious spectral effects that might have been introduced by the instrument setup and not the samples, such as slight changes in ambient lighting or slight movements of the scanning instrument in three-dimensional space during and between scans.

#### 3.2.3. Calibration and Validation Model Design

Partial least squares (PLS) regression analyses were run for datasets that were pre-processed in the preceding section using UCal 4^TM^ Custom Calibration Software. The calibration model design relied on data previously obtained as reference values for lipid content in the scanned pea samples obtained by solvent extraction using hexane-isopropanol (3:2, *v*/*v*) in a modified version of a method described previously [4]. In the absence of an independent validation sample set due to the small sample size available for this study, a cross-validation method was used for validating the calibration model. A leave-one-out cross-validation (LOOCV) was performed to generate the standard error of cross-validation (SECV) statistics, and each sample was used as a singular test set against the model built from the remaining training set.

## 4. Conclusions

Traditional methods for lipid quantification require several grams of sample, costly extraction solvents, and are time-consuming. The results showed that NMR and NIR are low-cost, rapid, and non-destructive methods for analyzing lipids in pea. With this study, we confirmed that NMR technology can be used for quantitative and qualitative analyses of the fatty acids present in oilseeds. These data show a high correlation between the NMR method and the traditional method in predicting the lipid content present in seeds. In comparing traditional extraction methods, however, an appropriate correction factor must be used with the measured NMR values. PLS regression analyses confirm the possible application of NIR analysis for the rapid determination of pea lipid content. With trends fitting models often close to R^2^ = 0.96, more samples and more plant cultivars will improve the robustness of the calibration model and an effective system may be developed to profile fatty acids and quantify lipids in various samples. Due to the versatility of NMR and NIR, future studies should explore the use of these technologies for the nutritional assessment of food products and the identification of adulterations or impurities.

## Figures and Tables

**Table 1:** Sample preparation comparison and percentage (% dry mass) of different fatty acids in field pea seeds.

Sample	Form	Linolenic Fatty Acid (%)	Linoleic Fatty Acid (%)	Oleic Fatty Acid (%)	Saturated Fatty Acid (%)
F1	Seed portion	26.62 ± 1.65 ^a^	23.57 ± 4.60 ^c^	44.49 ± 3.86 ^a^	5.32 ± 4.26 ^b^
	Ground	24.56 ± 0.56 ^a^	20.28 ± 5.07 ^c^	45.64 ± 5.52 ^a^	9.51 ± 0.90 ^b^
112351	Seed portion	23.84 ± 0.56 ^ab^	31.42 ± 1.42 ^b^	32.33 ± 0.65 ^bc^	12.42 ± 1.42 ^b^
	Ground	22.35 ± 2.69 ^ab^	30.81 ± 0.77 ^b^	34.89 ± 0.54 ^bc^	11.96 ± 2.63 ^b^
F2	Seed portion	22.29 ± 1.15 ^b^	34.16 ± 2.40 ^ab^	31.98 ± 1.10 ^bc^	11.56 ± 3.05 ^b^
	Ground	22.95 ± 2.15 ^b^	36.50 ± 3.60 ^ab^	29.28 ± 2.31 ^bc^	11.28 ± 0.20 ^b^
42819	Seed portion	23.42 ± 0.52 ^ab^	43.98 ± 7.73 ^a^	21.71 ± 7.72 ^c^	10.89 ± 0.21 ^b^
	Ground	23.86 ± 0.26 ^ab^	35.79 ± 3.45 ^a^	30.59 ± 3.77 ^c^	9.76 ± 0.79 ^b^
43016	Seed portion	20.88 ± 0.86 ^b^	35.30 ± 5.24 ^ab^	34.01 ± 4.90 ^bc^	9.81 ± 0.69 ^b^
	Ground	21.47 ± 0.92 ^b^	35.78 ± 5.32 ^ab^	32.29 ± 6.18 ^bc^	10.46 ± 0.45 ^b^
29600	Seed portion	23.27 ± 1.35 ^ab^	31.13 ± 3.05 ^b^	40.99 ± 3.42 ^ab^	4.61 ± 2.14 ^b^
	Ground	22.85 ± 1.61 ^ab^	28.97 ± 7.28 ^b^	41.03 ± 7.25 ^ab^	7.15 ± 1.25 ^b^
45760	Seed portion	21.24 ± 1.64 ^b^	36.63 ± 1.33 ^ab^	31.37 ± 1.30 ^bc^	10.76 ± 1.63 ^b^
	Ground	20.81 ± 2.72 ^b^	34.81 ± 3.13 ^ab^	31.71 ± 3.81 ^bc^	12.66 ± 0.94 ^b^
29579	Seed portion	24.08 ± 0.58 ^ab^	20.19 ± 0.52 ^c^	44.63 ± 3.75 ^a^	11.09 ± 3.24 ^b^
	Ground	22.61 ± 2.85 ^ab^	13.89 ± 3.45 ^c^	55.42 ± 1.17 ^a^	8.08 ± 4.74 ^b^
29526	Seed portion	25.52 ± 1.04 ^a^	21.00 ± 7.40 ^c^	25.24 ± 3.57 ^bc^	28.24 ± 4.50 ^a^
	Ground	25.45 ± 7.46 ^a^	18.42 ± 7.85 ^c^	34.37 ± 3.52 ^bc^	21.76 ± 2.83 ^a^

Values in the same column with the same letter superscript are not significantly different (*p* < 0.05). Values are the means of three replicates ± standard deviation.

**Table 2:** Comparative table of the actual total fatty acid (% dry mass) vs the corrected total fatty acid (% dry mass) recorded for soybean and field pea seed accessions.

Samples	Actual NMR Data	Khodapanahi et al. (2012)		Kalia (2016)		Ossowski (2017)	
		Actual Soxhlet (Butanol)	Corrected NMR	Actual Soxhlet (Hexane-Isopropanol)	Corrected NMR	Actual Soxhlet (Petroleum Ether)	Corrected NMR
Soybean	4.60 ± 0.23 b	13.90	14.17	NR	NR	NR	NR
F1	0.80 ± 0.06 a	NR	NR	NR	NR	NR	NR
112351	0.67 ± 0.05 a	1.60	2.06	1.40	1.32	0.61	0.57
F2	1.09 ± 0.05 a	NR	NR	NR	NR	NR	NR
42819	0.77 ± 0.10 a	2.50	2.37	1.70	1.52	0.67	0.65
43016	1.09± 0.09 a	3.60	3.36	2.20	2.15	NR	NR
29600	1.12 ± 0.08 a	2.60	3.45	1.70	2.21	NR	NR
45760	1.07 ± 0.12 a	3.50	3.30	2.10	2.11	1.05	0.91
29579	1.02 ± 0.06 a	3.70	3.14	2.60	2.01	0.74	0.87
29526	0.93 ± 0.10 a	3.10	2.86	1.30	1.83	0.69	0.79

Columns followed by the same letter are not significantly different (*p* < 0.05). Values represent the means of three replicates ± standard deviation. NR: not reported.

**Table 3:** PLS regression model cross validation data.

Pre-Processing Step(s)	Factors	R^2^	SECV	Reference Std. Dev.	RPD
[a]	4	0.96	0.13	0.50	3.97
[b]	3	0.98	0.11	0.50	4.62
[b] [c]	4	0.99	0.11	0.50	4.62

[a] No math treatment; [b] Standard normal variate and detrend treatment, forward derivatization (1st order); [c] Multiplicative Scattering Correction; SECV-Standard of error for cross-validation; RPD-Residual predictive deviation.

## Data Availability

Not applicable.

## Supplementary Materials

The following are available online, Table S1. Germination rate of pea seeds, Table S2. Average integral values of the peaks used for fatty acid quantification, Figure S1. 1H NMR spectrum of the pea seed, and Figure S2. Detailed view of the contact probe, light source, sample, and turntable sample trya for NIR analyses. Figure S3. NIR spectra from six pea cultivars.

## Sample Availability

Samples of the compounds are available from the authors.
