# Supplementary material for: Development of a Nuclear Magnetic Resonance Method and a Near Infrared Calibration Model for the Rapid Determination of Lipid Content in the Field Pea (Pisum sativum)
Source: Molecules. 2022 Mar 2;27(5):1642. doi: 10.3390/molecules27051642 (PMC8911919; doi:10.3390/molecules27051642)
Supplement: Supplementary file 1 [file molecules-27-01642-s001.zip › molecules-1598976-supplementary.pdf]

Table S1. Germination rate of pea seeds

Variety	Number of seeds planted	Number of germinated seeds	Germination rate (100%)
F1	3	3	100
112351	3	3	100
F2	3	3	100
42819	3	3	100
43016	3	3	100
29600	3	3	100
45760	3	3	100
29579	3	3	100
29526	3	3	100

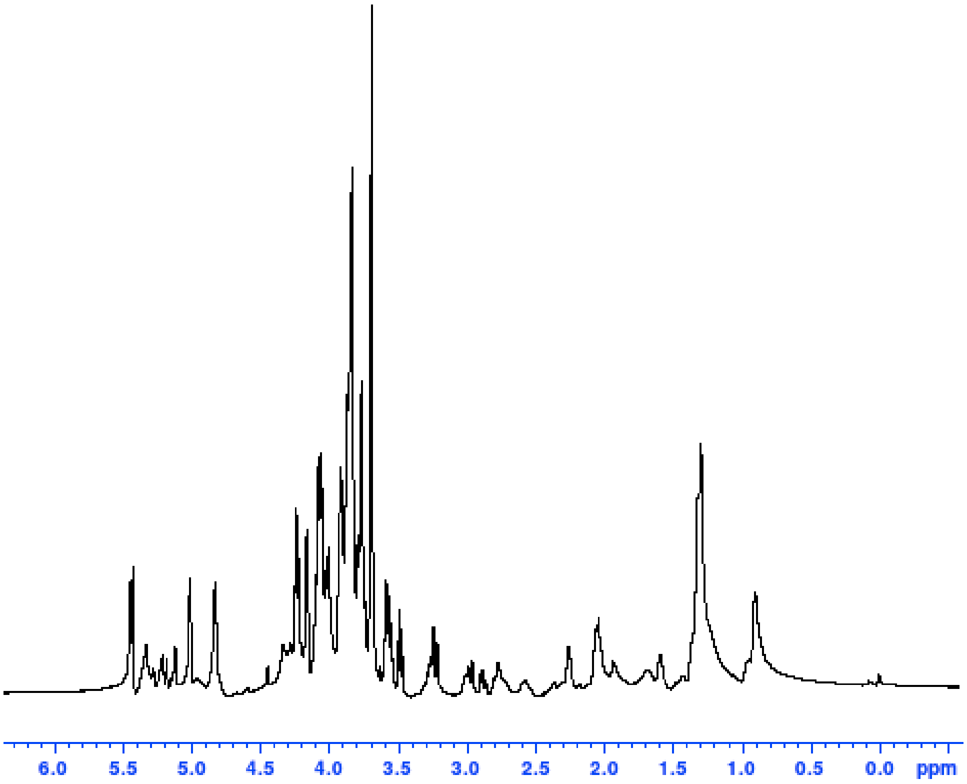

Figure S1. ^{1}H NMR spectrum of the pea seed.

Table S2. Average integral values of the peaks used for fatty acid quantification

Sample		H	β	α_1	$\alpha 2$	A	B	C	D	G	E	F
	Pea oil	7.908	0.935	1.589	1.712	4.181	5.913	9.954	6.540	50.618	1.610	9.038
F1	Seed pieces	0.072	0.048	0.062	0.103	0.043	0.056	0.106	0.052	0.200	0.023	0.062
	Ground	0.069	0.041	0.053	0.090	0.038	0.055	0.099	0.050	0.199	0.020	0.063
112351	Seed pieces	0.056	0.036	0.036	0.075	0.030	0.038	0.066	0.035	0.159	0.014	0.045
	Ground	0.050	0.029	0.034	0.076	0.023	0.034	0.063	0.034	0.158	0.015	0.046
F2	Seed pieces	0.100	0.048	0.043	0.101	0.049	0.062	0.111	0.057	0.350	0.025	0.087
	Ground	0.078	0.039	0.035	0.073	0.036	0.046	0.081	0.044	0.240	0.020	0.064
42819	Seed pieces	0.076	0.043	0.047	0.108	0.044	0.048	0.086	0.045	0.221	0.019	0.061
	Ground	0.073	0.042	0.045	0.096	0.036	0.043	0.077	0.041	0.206	0.018	0.056
43016	Seed pieces	0.098	0.047	0.055	0.124	0.048	0.062	0.112	0.061	0.375	0.023	0.086
	Ground	0.100	0.050	0.060	0.133	0.049	0.062	0.111	0.061	0.355	0.023	0.084
29600	Seed pieces	0.096	0.048	0.060	0.130	0.050	0.064	0.123	0.064	0.326	0.027	0.089
	Ground	0.104	0.053	0.070	0.142	0.051	0.068	0.127	0.066	0.353	0.027	0.092
45760	Seed pieces	0.102	0.053	0.073	0.148	0.044	0.056	0.100	0.056	0.325	0.019	0.071
	Ground	0.100	0.053	0.067	0.140	0.048	0.060	0.106	0.058	0.307	0.024	0.081
29579	Seed pieces	0.061	0.035	0.044	0.085	0.031	0.041	0.074	0.039	0.195	0.012	0.042
	Ground	0.061	0.034	0.041	0.077	0.033	0.045	0.081	0.042	0.209	0.015	0.051
29526	Seed pieces	0.067	0.029	0.048	0.100	0.023	0.039	0.072	0.037	0.186	0.016	0.045
	Ground	0.104	0.055	0.067	0.108	0.041	0.059	0.094	0.055	0.209	0.024	0.058

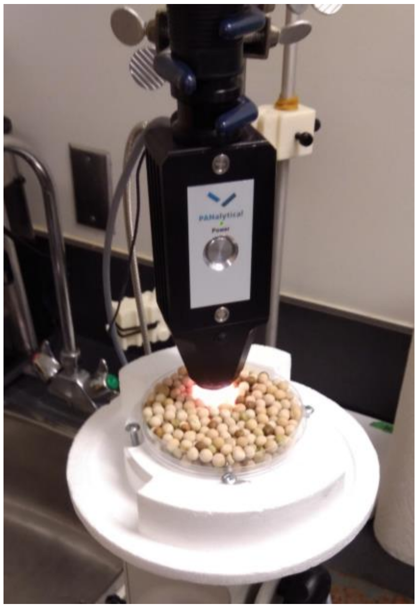

Figure S2. Detailed view of the contact probe, light source, sample, and turntable sample tray for NIR analyses.

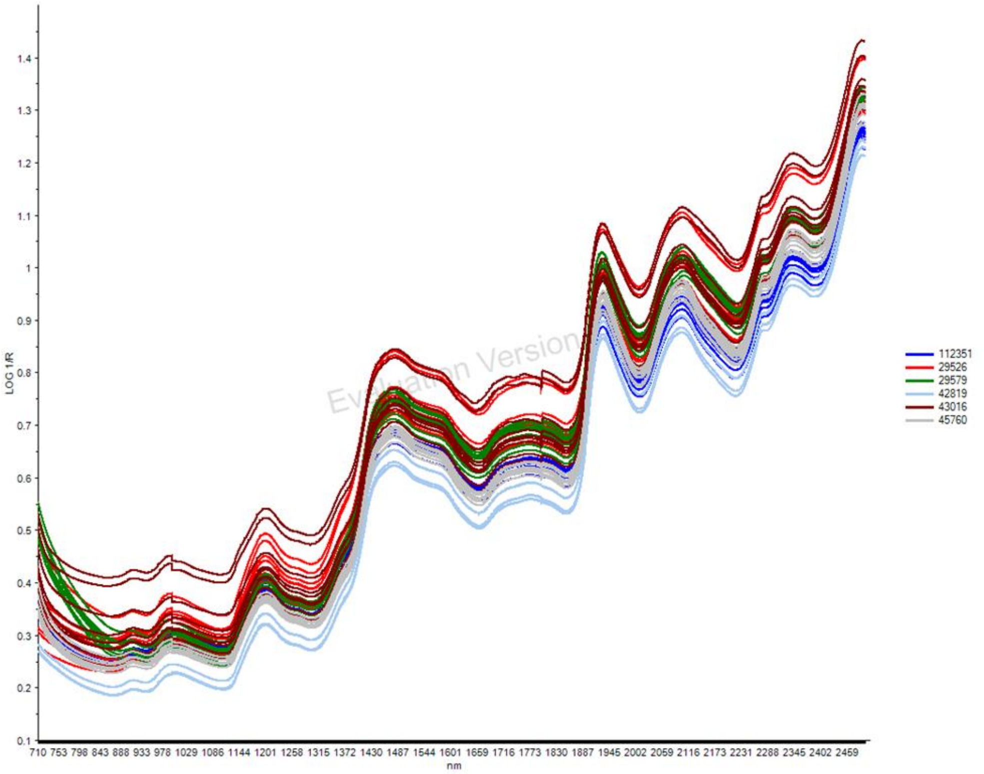

Figure S3. NIR spectra from six pea cultivars.
